# Registered report: Biomechanical remodeling of the microenvironment by stromal caveolin-1 favors tumor invasion and metastasis

**DOI:** 10.7554/eLife.04796

**Published:** 2015-07-16

**Authors:** Steven Fiering, Lay-Hong Ang, Judith Lacoste, Tim D Smith, Erin Griner, Reproducibility Project: Cancer Biology, Elizabeth Iorns

**Affiliations:** Science Exchange, Palo Alto, California; Mendeley, London, United Kingdom; Science Exchange, Palo Alto, California; Science Exchange, Palo Alto, California; Center for Open Science, Charlottesville, Virginia; 1 Transgenics and Genetic Constructs Shared Resource Center, Dartmouth University, Lebanon, United States; 2 Confocal Imaging Core, Harvard Medical School, Boston, United States; 3 MIA Cellavie Inc., Montreal, Canada; 4 University of California, Irvine, Irvine, United States; 5 University of Virginia, Charlottesville, United States; University College London, United Kingdom

**Keywords:** Reproducibility Project: Cancer Biology, methodology, metastasis, biochemical remodeling, tumor microenvironment, human, mouse

## Abstract

The Reproducibility Project: Cancer Biology seeks to address growing concerns about reproducibility in scientific research by conducting replicating selected results from a number of high-profile papers in the field of cancer biology. The papers, which were published between 2010 and 2012 were selected on the basis of citations and Altimetric scores (Errington et al., 2014). This Registered report describes the proposed replication plan of key experiments from ‘Biomechanical remodeling of the microenvironment by stromal caveolin-1 favors tumor invasion and metastasis’ by Goetz and colleagues, published in *Cell* in 2011 (Goetz et al., 2011). The key experiments being replicated are those reported in Figures 7C (a-d), Supplemental Figure S2A, and Supplemental Figure S7C (a-c) (Goetz et al., 2011). In these experiments, which are a subset of all the experiments reported in the original publication, Goetz and colleagues show in a subcutaneous xenograft model that stromal caveolin-1 remodels the intratumoral microenvironment, which is correlated with increased metastasis formation. The Reproducibility Project: Cancer Biology is a collaboration between the Center for Open Science and Science Exchange and the results of the replications will be published in *eLife*.

**DOI:** http://dx.doi.org/10.7554/eLife.04796.001

## Introduction

The importance of the tumor microenvironment in cancer progression is well established. Cancer-associated fibroblasts (CAFs) are one subset of cells found in the tumor stroma that help to regulate tumor progression and metastasis through several mechanisms such as remodeling of the extracellular matrix, secretion of growth factors and chemokines, and regulation of epithelial-to-mesenchymal transition (Kalluri and Zeisberg, 2006; Cirri and Chiarugi, 2011). Caveolin-1 (Cav1) is an essential component of caveolae and regulator of lipid raft formation that plays an important role in tumor progression (Goetz et al., 2008; Sotgia et al., 2012). However, the precise role of Cav1 in tumor progression appears to be unclear, with studies showing both increased and decreased expression in various types of cancer (Parton and del Pozo, 2013). One possible model is a general trend in which Cav1 appears to act as a tumor suppressor at early stages of cancer progression, but is up-regulated in several multidrug-resistant and metastatic cancer cell lines and human tumor specimens, positively correlating with tumor stage and grade in numerous cancer types (Shatz and Liscovitch, 2008). Part of this variability could stem from Cav1 expression in stromal rather than tumor cells. For instance, loss of Cav1 function in stromal cells of various organs leads to benign stromal lesions responsible for abnormal growth and differentiation of the epithelium and to dramatic reductions in life span (Yang et al., 2008). Goetz and colleagues, reported that Cav1 expression in fibroblasts remodeled the extracellular matrix to regulate cell shape and to stimulate migration and invasion of cancer cells in vitro, and promote tumor growth and metastasis in vivo in a p190RhoGAP dependent manner (Goetz et al., 2011).

In order to specifically assess the role of Cav1 in the tumor stroma on intratumoral microenvironment remodeling and metastasis, Goetz and colleagues utilized a subcutaneous xenograft model in which primary mouse embryonic fibroblasts (pMEFs) derived from either wild type (WT) or Cav1 knock out animals (Cav1 KO) were coinjected subcutaneously with luminescent LM-4175 metastatic breast tumor cells (Minn et al., 2005) with Matrigel into nude mice (Goetz et al., 2011). Figure 7Ca presents a graphical illustration of the experimental procedure. Primary tumor growth and metastatic tumor growth was assessed by bioluminescent imaging in vivo or ex vivo in extracted organs. Further, immunostaining of primary tumors was utilized to analyze the fiber alignment and organization of the tumor stroma in the presence of WT or Cav1 KO pMEFs, as alignment of collagen fibers in the tumor microenvironment has been shown to enhance tumor cell migration and invasion (Amatangelo et al., 2005; Provenzano et al., 2006). It is important to note that the authors also performed shRNA knock down of p190RhoGAP in Cav1 KO pMEFs to show that the effects of Cav1 KO on metastasis could be reversed by knock down of p190RhoGAP in Cav1 KO pMEFs. This replication study will only address the effects of Cav1 expression in tumor stroma and remodeling of the tumor microenvironment matrix, and will not replicate the effects of p190RhoGAP in this model.

These experiments utilize isolated WT or Cav1 KO pMEFs, which are generated in Protocol 1. The loss of Cav1 in the Cav1 KO pMEFs (Figure 7Ca), lead to an increase in smooth muscle actin (SMA) compared to Cav1 WT pMEFs, which is an indicator of increased activation and extra cellular matrix (ECM) remodeling capabilities of the generated pMEFs (Supplemental Figure S2A) (Goetz et al., 2011). These pMEFs were used in a subcutaneous tumorigenicity assay, with representative bioluminescent images of primary tumors in vivo or ex vivo in extracted organs reported in Figure 7Cc. Bioluminescence of the primary tumors or metastatic foci in each organ was quantified and shown in Figure 7Cb or Supplemental Figure S7Ca. Goetz and colleagues evaluated metastatic tumor growth where they reported an increase in metastatic foci when tumor cells were coinjected with WT pMEFs compared to tumor cells alone or tumor cells coinjected with Cav1 KO pMEFs (Goetz et al., 2011). While not the main focus of this experiment they did not observe a difference in primary tumor growth between these conditions (Goetz et al., 2011). This experiment is important to replicate because it tests the central tenet of the paper, namely that Cav1 expression in stromal fibroblasts contributes to increased metastasis of tumors. This experiment is replicated in Protocol 3.

Although there was no observed difference in primary tumor growth when Cav1 was absent in the tumoral stroma specifically, a decrease in primary tumor growth was observed when the whole mammary gland was deficient for Cav1 in mammary gland allografts and xenografts (Goetz et al., 2011). In a related study, subcutaneous injection of B16 melanoma cells in Cav1 KO mice resulted in a reduced tumor growth compared to injection of tumor cells in WT mice (Chang et al., 2009). Another study, which focused on the size of the primary tumors opposed to metastasis, reported that intradermal coinjection of nude mice with B16F10 melanoma cells and Cav1 KO neonatal dermal fibroblasts increased primary tumor growth when compared to coinjection of tumor cells with WT fibroblasts (Capozza et al., 2012). While no known direct replications of the original study have been reported, several studies have assessed the role of stromal Cav1 expression in different types of tumors, with some studies reporting high Cav1 expression correlated with poor patient survival (Linke et al., 2010; Goetz et al., 2011; Righi et al., 2014), and others reporting low Cav1 expression in the stroma negatively correlated with survival (Simpkins et al., 2012; Ma et al., 2013; Zhao et al., 2013; Ren et al., 2014).

Figure 7Cc and Supplemental Figure S7Cb show representative images of tumor sections immunostained for fibronectin, the CAF marker SMA, and nuclei. Goetz and colleagues showed that fibronectin fiber alignment increased in the presence of Cav1 (Goetz et al., 2011). Supplemental Figure S7Cc shows the results of quantification of fibronectin and SMA fiber alignment as well as cell shape. Goetz and colleagues also show in Figure 7Cd that metastasis correlates with the degree of fibronectin fiber alignment in the tumor stroma. These figures are important to replicate as they show that Cav1 expression in fibroblasts contributes to remodeling of the tumor microenvironment in vivo, and that this remodeling correlates with the amount of metastasis. These experiments are replicated in Protocol 4.

## Materials and methods

### Protocol 1: isolation of Cav1 WT and Cav1 KO primary MEFs

This experiment describes the isolation of primary MEFs (pMEFs) that will subsequently be used in Protocols 2 and 3.

#### Sampling

■ Each experiment has 2 cohorts:
  ○ Cohort 1: Cav1-WT embryos.
  ○ Cohort 2: Cav1-KO embryos.
■ Experiment performed with three pregnant females in each cohort to ensure enough pMEFs are obtained.
  ○ Power calculations are not applicable.

#### Materials and reagents

**Table:** 

Reagent	Type	Manufacturer	Catalog #	Comments
6–8 week old B6129SF2/J mice (Cav1 WT)	Animal model	Jackson laboratory	101045	1 male and 3 females for breeding
6–8 week old CAV1<tm1mls>/J mice (Cav1 KO)	Animal model	Jackson laboratory	004585	1 male and 3 females for breeding
Ethanol	Chemical	Sigma–Aldrich	E7023	Original not specified
PBS, without MgCl_2_ and CaCl_2_	Buffer	Sigma–Aldrich	D8537	Original not specified
0.05% trypsin/0.48 mM EDTA	Cell culture	Sigma–Aldrich	T3924	Original not specified
50 ml tubes	Labware	Sigma–Aldrich	CLS430290	Original not specified
Dulbecco's modified Eagle's medium—low glucose, without L-glutamine	Cell culture	Sigma–Aldrich	D5546	Original not specified
Fetal bovine serum	Cell culture	Sigma–Aldrich	F0392	Original not specified
L-glutamine	Cell culture	Sigma–Aldrich	G7513	Original not specified
100× Pen/Strep	Cell culture	Sigma–Aldrich	P4333	Original not specified
150 mm tissue culture dishes	Labware	Sigma–Aldrich	CLS430599	Original not specified
100 mm tissue culture dishes	Labware	Sigma–Aldrich	CLS430167	Original not specified
60 mm tissue culture dishes	Labware	Sigma–Aldrich	CLS430166	Original not specified

#### Procedure

##### Notes

- This protocol contains information described in Cerezo et al. (2009), Razani et al. (2001), and Kamijo et al. (1997).
- pMEFs grown in complete DMEM: DMEM supplemented with 10% FBS, 2 mM L-glutamine, 100 U/ml penicillin and 100 µg/ml streptomycin at 37°C in a humidified atmosphere at 5% CO_2_.

1. Sacrifice E14.5 pregnant Cav1WT (B6129SF2/J) and Cav1KO (CAV1<tm1mls>/J) mouse by cervical dislocation.
2. Dissect uterine horns and transfer to tube with ice-cold PBS to a tissue culture hood.
3. Transfer uterine horns to a 100 mm tissue culture dish; separate embryos from and discard placenta and embryonic sac.
4. For each embryo, place in a dry 100 mm plate, dissect and discard head and red organs.
5. Mince with sterile scissors or a sterile razor blade until homogeneous and pipettable.
6. Add 1 ml 0.05% trypsin/0.53 mM EDTA per embryo, disaggregate further by pipetting, and incubate 5 min at 37°C.
7. Plate 1 ml of cells/trypsin into 150 mm tissue culture dishes (one dish per embryo), and try to further disaggregate by pipetting.
  a. Ensure medium is pre-warmed before adding cells.
  b. These are ‘passage 1’ cells.
8. Repeat the disaggregation with the pipette every 10 min, 3 or 4 times (steps 6–7).
9. Change the medium after 24 hr.
10. On day 3 and every third day thereafter, passage 3 × 10^5^ cells per 60 mm dish.
  a. Use pMEFs before passage 5.
11. Use pMEFs in further experiments below.
  a. Western blot analysis of Cav1 and SMA levels (Protocol 2).
  b. Subcutaneous tumorigenicity assay (Protocol 3).
12. Repeat for each pregnant mouse.

##### Deliverables

■ Data to be collected:
  ○ Mouse health records (gender of mice, age of embryos when sacrificed).
■ Sample delivered for further analysis:
  ○ pMEFs derived from Cav1WT and Cav1KO mouse embryos (Protocols 2 & 3).

#### Confirmatory analysis plan

This protocol will not perform any statistical tests.

#### Known differences from the original study

All known differences, if any, are listed in the materials and reagents section above with the originally used item listed in the comments section. The comments section also lists if the source of original item was not specified. All differences have the same capabilities as the original and are not expected to alter the experimental design.

#### Provisions for quality control

All of the raw data, will be uploaded to the project page on the OSF (https://osf.io/7yqmp) and made publically available.

### Protocol 2: assessment of Cav1 and SMA levels in pMEFs by western blot

This experiment assesses the protein levels of Cav1 to ensure the Cav1 KO pMEFs generated are actually knockout for Cav1. Additionally, SMA is assessed to determine if levels in Cav1 KO pMEFs are increased over Cav1 WT pMEFs, which indicate increased activation and ECM remodeling capabilities of the generated pMEFs. It is similar to the experiments reported in Figure 7Ca and Supplemental Figure S2A.

#### Sampling

- Each experiment has two cohorts:
  ○ Cohort 1: Cav1-WT pMEFs.
  ○ Cohort 2: Cav1-KO pMEFs.
- Each clone of pMEFs will be assessed for the following markers:
  ○ Cav1.
  ○ SMA.
  ○ γ-tubulin.
- Experiment will be conducted once and used to assess which clones will be utilized.
  ○ Power calculations are not applicable.

#### Materials and reagents

**Table:** 

Reagent	Type	Manufacturer	Catalog #	Comments
60 mm tissue culture dishes	Labware	Sigma–Aldrich	CLS430166	
PBS, without MgCl_2_ and CaCl_2_	Buffer	Sigma–Aldrich	D8537	Original not specified
RIPA lysis buffer	Buffer	Specific brand information will be left up to the discretion of the replicating lab and recorded later		
Cell scraper	Labware			
Refrigerated centrifuge	Equipment			
Bradford assay	Reporter assay			
Molecular weight marker	Western materials			
6× SDS-PAGE sample buffer	Buffer	Specific brand information used to make these reagents will be left up to the discretion of the replication lab and recorded later		
SDS-PAGE gel	Western materials			
Tris-glycine SDS-PAGE running buffer	Buffer			
Electrotransfer buffer	Buffer			
Ponceau S stain	Stain			
TBS buffer	Buffer			
PVDF membrane	Western materials	Specific brand information will be left up to the discretion of the replicating lab and recorded later		
Tween-20	Chemical			
Non-fat dry milk	Western materials			
ECL chemiluminescent reagent	Western materials			
Mouse anti-Cav1 (clone 2297)	Antibodies	BD Biosciences	610406	Original clone/catalog# unspecified
Mouse anti-α-smooth muscle actin (clone 1A4)	Antibodies	Sigma–Aldrich	A5228	
Mouse anti-γ-tubulin (clone GTU-88)	Antibodies	Sigma–Aldrich	T6557	Original clone/catalog # unspecified
Goat anti-mouse-HRP	Antibodies	Life Sciences	32,430	

#### Procedure

##### Note

- pMEFs are generated in Protocol 1.
- pMEFs maintained in: DMEM supplemented with 10% FBS, 2 mM L-glutamine, 100 U/ml penicillin and 100 µg/ml streptomycin at 37°C in a humidified atmosphere at 5% CO_2_.

1. Prepare each clone of pMEFs for lysis.
  a. Wash cells with ice-cold PBS and remove excess PBS.
  b. Add cold RIPA buffer to cells (use 1 ml of buffer per 60 mm dish).
  c. Keep on ice for 5 min, swirling plate occasionally for uniform spreading.
  d. Collect lysate with cell scraper and transfer to microcentrifuge tube.
  e. Centrifuge samples at ∼14,000×*g* for 15 min at 4°C.
  f. Transfer supernatant to new tube.
2. Determine protein concentration using Bradford assay following manufacturer's instructions and a BSA standard curve.
3. Adjust protein concentration and prepare up to 70 µg/lane of total cell lysate by adding 6× SDS-PAGE sample buffer and heating to 100°C for 5 min.
4. Separate samples and molecular weight marker by SDS-PAGE gel electrophoresis in 1× tris-glycine SDS buffer following replicating lab's protocol. Run at 100V through the stacking part of the gel and up to 200 V after the proteins have migrated through the resolving gel. Allow migration to continue until the blue dye front is at the bottom of the gel, but has not migrated off.
  a. Include Cav1 WT and Cav1 KO clones on same gel to allow comparison.
5. Transfer gel to a PVDF membrane, following replicating lab's transfer procedure.
6. After the transfer, stain the membrane with Ponceau S to visualize the transferred protein. Image membrane, than destain in ddH_2_O and rinse with TBS buffer.
7. Incubate membrane with 5% non-fat dry milk in TBST buffer.
  a. TBST buffer: TBS with 0.1% Tween-20.
8. Probe membrane with the following primary antibodies diluted in 5% non-fat dry milk in TBST buffer:
  a. mouse anti-Cav1; use at 1:1000; 21 kDa.
  b. mouse anti-SMA; use at 1:1000; 42 kDa.
  c. mouse anti-γ-tubulin; use at 1:1000; 48 kDa.
9. Wash membrane in TBST buffer.
10. Detect primary antibodies with the following secondary antibody diluted in 5% non-fat dry milk in TBST buffer:
  a. anti-mouse-HRP; use at 1:5000 to 1:10,000.
11. Wash membrane in TBST buffer.
12. Detect signal with ECL reagent following manufacturer's instructions.
13. Image the entire membrane including molecular weight ladder.
14. Quantify signal intensity.
  a. For each antibody subtract background intensity from values and then divide by the γ-tubulin loading control.
  b. Calculate the normalized SMA levels for all clones.
  c. Confirm absence of Cav1 protein in all Cav1 KO pMEFs.
15. Exclude any clones that do not display a presence (Cav1 WT pMEFs) or absence (Cav1 KO pMEFs) of Cav1. Exclude any clones that do not have an increase in SMA expression with a loss of Cav1 (Cav1 KO pMEFs compared to Cav1 WT pMEFs).
16. Use remaining pMEF clones in further experiments before passage 5:
  a. Subcutaneous tumorigenicity assay (Protocol 3).

#### Deliverables

■ Data to be collected:
  ○ Images of probed membranes (full images with ladder).
  ○ Raw and quantifed signal intensities normalized for γ-tubulin loading and total protein levels.
■ Sample delivered for further analysis:
  ○ Cav1 WT and Cav1 KO pMEFs that are included for further use (Protocol 3).

#### Confirmatory analysis plan

This protocol will not perform any statistical tests.

#### Known differences from original study

The replicating lab western blot protocol will be used. All known differences, if any, are listed in the materials and reagents section above with the originally used item listed in the comments section. The comments section also lists if the source of original item was not specified. All differences have the same capabilities as the original and are not expected to alter the experimental design.

#### Provisions for quality control

Transfer quality will be assured by Ponceau staining. All of the raw data, will be uploaded to the project page on the OSF (https://osf.io/7yqmp) and made publically available. This experiment is also the quality control for the pMEFs generated in Protocol 1 that will be utilized in Protocol 3 to assess Cav1 status and ECM remodeling capabilities.

### Protocol 3: subcutaneous tumorigenicity assay of tumor cells co-injected into athymic nude mice with pMEFs

This experiment tests the contribution of Cav1 expression in pMEFs on tumorigenicity and metastasis of breast cancer cells. pMEFs derived from WT or Cav1 KO animals are co-injected with LM-4175 breast cancer cells in a Matrigel plug subcutaneously in nude mice and tumor growth and metastasis are monitored by bioluminescent imaging. It is a replication of the experiment reported in Figure 7Cb and Supplemental Figure S7Ca.

#### Sampling

■ Experiment has 3 cohorts:
  ○ Cohort 1: LM-4175 cells alone.
  ○ Cohort 2: LM-4175 cells co-injected with Cav1 WT pMEFs.
  ○ Cohort 3: LM-4175 cells co-injected with Cav1 KO pMEFs.
■ Experiment will analyze the following number of mice per cohort for a minimum power of 80%:
  ○ See Power calculations section for details.
    - Cohort 1: 7 mice.
    - Cohort 2: 21 mice.
    - Cohort 3: 21 mice.
■ To account for unexpected euthanasia of mice before the end of the experiment, 20% more mice were added to ensure the needed number of mice survive each cohort:
  ○ Cohort 1: 9 mice.
  ○ Cohort 2: 26 mice.
  ○ Cohort 3: 26 mice.

#### Materials and reagents

**Table:** 

Reagent	Type	Manufacturer	Catalog #	Comments
LM-4175 cells expressing HSV-tk1-GFP-Fluc	Cell line	Original lab	n/a	From original lab
Dulbecco's modified Eagle's medium—low glucose, without L-glutamine	Cell culture	Sigma–Aldrich	D5546	Original not specified
PBS, without MgCl_2_ and CaCl_2_	Buffer	Sigma–Aldrich	D8537	Original not specified
0.05% trypsin/0.48 mM EDTA	Cell culture	Sigma–Aldrich	T3924	Original not specified
50 ml tubes	Labware	Sigma–Aldrich	CLS430290	Original not specified
Fetal bovine serum	Cell culture	Sigma–Aldrich	F0392	Original not specified
L-glutamine	Cell culture	Sigma–Aldrich	G7513	Original not specified
100× pen/Strep	Cell culture	Sigma–Aldrich	P4333	Original not specified
100 mm tissue culture dishes	Labware	Sigma–Aldrich	CLS430167	Original not specified
60 mm tissue culture dishes	Labware	Sigma–Aldrich	CLS430166	Original not specified
Matrigel matrix	Cell culture	Corning	356234	Original from Becton Dickinson
8–10 week old female athymic nude mice	Animal model	Harlan	Hsd:Athymic Nude-*Foxn1*^*nu*^	Mice should be acclimated for 2 weeks before the start of experiment
Ketamine	Chemical	Specific brand information will be left up to the discretion of the replicating lab and recorded later		
Xylazine	Chemical			
25 G needle	Labware	Sigma–Aldrich	Z192406	Original not specified
1 ml syringe	Labware	Sigma–Aldrich	Z192090	Original not specified
VivoGlo Luciferin	Reporter assay	Promega	P1042	Original not specified
IVIS Imaging System	Instrument	PerkinElmer	200 Series	
Living Image software	Software	PerkinElmer	Version 4.3.1	
O.C.T. compound (Tissue-Tek)	Buffer	VWR	25,608-930	Original not specified

#### Procedure

##### Note

- pMEFs are generated in Protocol 1 with inclusion/exclusion criteria determined in Protocol 2.
- pMEFs maintained in: DMEM supplemented with 10% FBS, 2 mM L-glutamine, 100 U/ml penicillin and 100 µg/ml streptomycin at 37°C in a humidified atmosphere at 5% CO_2_.
- LM-4175 tumor cells express HSV-tk1-GFP-Fluc and will be sent for mycoplasma testing and STR profiling as well as screened against a Rodent Pathogen Panel.
- LM-4175 maintained in: normal glucose DMEM supplemented with 10% FBS at 37°C in a humidified atmosphere at 5% CO_2_.
- Athymic nude mice should be 8–10 weeks old when they arrive and about 10–12 weeks old when injected with cells.

1. Cell preparation for injection:
  a. LM-4175 tumor cells alone:
    i. Prepare 1 × 10^7^ cells/ml of LM-4175 tumor cells in sterile chilled PBS.
      1. You will need 0.1 ml (1 × 10^6^ cells) per injection.
      2. Adjust cell mixture accordingly for needed number of injections.
    ii. Mix cell suspension.
  b. LM-4175 tumor cells with pMEFs.
    i. Prepare 2 × 10^7^ cells/ml of LM-4175 tumor cells in sterile chilled PBS.
    ii. Prepare 2 × 10^7^ cells/ml of Cav1 WT pMEFs, or Cav1 KO pMEFs in sterile chilled PBS.
    iii. Mix the two suspensions together to form a 1:1 mixture.
      1. You will need 0.1 ml (2 × 10^6^ cells) per injection.
      2. Adjust cell mixture accordingly for needed number of injections.
  c. Using well-chilled tubes and pipette tips, mix each final cell suspension with an equal amount (0.1 ml) of Matrigel and store on ice.
2. Anesthetize 10–12 week old female athymic nude mice.
  a. House mice under pathogen-free conditions and give autoclaved food and water *ad libitum.*
  b. Anesthetize with 100 mg/kg ketamine and 10 mg/kg xylazine.
3. Using a 25 G needle and 1 ml syringe, aspirate 0.2 ml of Matrigel/cell suspension and inject subcutaneously at the prepared site.
  a. During injection keep the mice warm by lying them on a warming plate designed for animal experiments.
  b. Stretch the abdominal cavity avoiding the formation of wrinkles.
  c. Inject Matrigel/cell suspension slowly by introducing the needle as parallel as possible to the skin. Ensure the Matrigel/cell suspension forms a visible bump under the skin.
  d. If injection enters the peritoneum, there will not be a bubble in the skin and instead of formation of one primary tumor there will be many intraperitoneal tumors. These mice should be excluded from the experiment and analysis.
    i. It is important not to take the needle out of the skin until the Matrigel has gelled. Gelification takes a very short time at body temperature (∼1 min).
4. Allow mice to recover and keep them warm until they wake up.
5. Maintain mice for 70 days.
  a. Measure tumor size twice weekly with precision calipers.
  b. Initial tumor formation is defined as the time when the tumor reaches a diameter of 3 mm. The tumor should never be greater than 10% body weight or exceed 20 mm in any one dimension. If ulceration or infection at the tumor site, or interference with eating or impairment of ambulation by the tumor occurs, the animals should be euthanized.
  c. Additional euthanasia criteria to ensure no animal suffering (when two or more of these criteria are detected, monitor the animals for 12 hr, if the condition does not improve, euthanize):
    i. Rapid or progressive weight loss (more than 10% in 2 weeks).
    ii. Debilitating diarrhea.
    iii. Dehydration/reduced skin turgor.
    iv. Edema.
    v. Sizable abdominal enlargement or ascites.
    vi. Hunched posture.
    vii. Lethargy.
    viii. Labored breathing, nasal discharge.
    ix. Bleeding from any orifice.
    x. Any condition interfering with daily activities for more than 2 hr (e.g., eating or drinking, ambulation, or elimination).
    xi. Excessive or prolonged hyperthermia or hypothermia.
6. At 70 days (or an earlier time point if the number of mice euthanized in step 5 compromise the ability to obtain enough mice for analysis), anesthetize each mouse and inject pairs of mice with 150 µl of 17.5 mg/ml luciferin solution intraperitoneally.
  a. Anesthetize with 100 mg/kg ketamine and 10 mg/kg xylazine.
  b. Prepare solution of luciferin according to manufacturer's instructions.
  c. Within each pairing of mice image, euthanize, dissect, re-image, and freeze tumors (steps 6–10) from mice from different cohorts in parallel (i.e., one from each of the three cohorts, or one each from cohort 2 and cohort 3) so variations during the procedure are equal across cohorts.
7. After 20 min post luciferin injection, place in IVIS Imaging System and take ventral views for photon flux quantification.
  a. To facilitate metastasis detection in axillary/brachial lymph nodes, secure front limbs with tape, and shield the lower portion of the animal to block bioluminescence from the primary tumors.
  b. Use extended exposures (0.2 s–20 s) for in vivo metastases detection.
  c. To detect well-defined in vivo metastases, shield lower portion of the animal to block bioluminescence from the primary tumors and bladder, and use a 20 s exposure.
8. Euthanize mouse and excise the primary tumor and extract organs.
  a. Before euthanasia, inject a second dose of luciferin solution—50 µl of 17.5 mg/ml luciferin solution intraperitoneally.
  b. Wait 20 min, euthanize mouse and quickly excise the primary tumor and extract the following organs to image for metastasis:
    i. Lymph nodes.
    ii. Spleen.
    iii. Lungs.
    iv. Liver.
    v. Intestines.
    vi. Kidneys.
9. Reimage organs ex vivo in IVIS imaging system.
  a. Place all organs in plastic dish (uncovered), with organs separated, for imaging.
  b. Reimage organs as quickly as possible after organ dissection.
  c. Acquire multiple exposure times to manually quantify every visible metastatic focus. (Recommend 1, 20, and 60 s exposures, with 2 min as longest exposure time).
  d. Small metastatic foci can be detected by adjusting the scale of photon flux in Living Image software (version 3.2).
10. Cut extracted primary tumors in half and freeze in O.C.T. compound for further analysis (Protocol 4). Randomly select the following number of tumors from each cohort:
  a. LM-4175 cells alone—4 tumors.
  b. LM-4175 cells co-injected with Cav1 WT pMEFs—7 tumors.
  c. LM-4175 cells co-injected with Cav1 KO pMEFs—7 tumors.

#### Deliverables

■ Data to be collected:
  ○ Passage of cells, particularly pMEFs, injected in each mouse.
  ○ Mouse health records (including if euthanasia is required and reason, date of euthanasia, and date of imaging if it is not 70 days).
  ○ Twice weekly tumor measurements.
  ○ All images of mice in vivo to detect primary tumor (compare to Figure 7Cc).
  ○ All images of excised organs (ex vivo) to detect metastatic foci (compare to Figure 7Cc).
  ○ Raw photon flux measurements of primary tumor and metastasis in vivo and metastatic foci ex vivo, including counts of metastatic foci from ex vivo images.
  ○ Graph of primary tumor growth (photon flux (P/s, ×10^10^) for all conditions (compare to Figure S7Ca).
  ○ Graph of metastatic foci (ex vivo bioluminescence) per mouse for all conditions. One graph for each organ type and for total organs (compare to Figure 7Cb).
■ Sample delivered for further analysis:
  ○ Preserved primary tumors for further analysis in Protocol 4.

#### Confirmatory analysis plan

This replication attempt will perform the following statistical analysis listed below:

■ Statistical analysis:
  ○ Wilcoxon–Mann Whitney test of total metastatic foci counts per mouse by ex vivo bioluminescence for the following comparisons with the Bonferroni correction:
    Note: in order to enable a direct comparison to how the original data was analyzed, uncorrected tests will also be performed.
    1. LM-4175 only to LM-4175 co-injected with Cav1 WT.
    2. LM-4175 only to LM-4175 co-injected with Cav1 KO.
    3. LM-4175 co-injected with Cav1 WT to LM-4175 co-injected with Cav1 KO.
  ○ Kruskal–Wallis test of primary tumor growth per mouse by in vivo bioluminescence for all conditions.
■ Meta-analysis of effect sizes:
  ○ Compute the effect sizes of each comparison, compare them against the reported effect size in the original paper and use a random effects meta-analytic approach to combine the original and replication effects, which will be presented as a forest plot.

#### Known differences from the original study

The replication attempt is not including the experimental condition of p190RhoGAP knockdown in Cav1 KO pMEFs. All known differences, if any, are listed in the materials and reagents section above with the originally used item listed in the comments section. The comments section also lists if the source of original item was not specified. All differences have the same capabilities as the original and are not expected to alter the experimental design.

#### Provisions for quality control

The pMEFs used in this protocol were assessed for Cav1 status and ECM remodeling capabilities (SMA levels) in Protocol 2. The cell lines used in this experiment will undergo STR profiling to confirm their identity and will be sent for mycoplasma testing to ensure there is no contamination. Additionally, cells will be screened against a Rodent Pathogen Panel to ensure no contamination prior to injection. Mice will be injected with luciferin, imaged, euthanized, dissected, re-imaged, and tumors frozen one animal from each cohort done in parallel to have variations during the procedure equal across cohorts. All of the raw data, including the image files and quantified metastatic foci, will be uploaded to the project page on the OSF (https://osf.io/7yqmp) and made publically available.

### Protocol 4: examining intratumoral fiber orientation and cell shape in tumor cells co-injected into nude mice with pMEFs

This experiment tests the effect of Cav1 expression in pMEFs on fiber orientation and cell shape within the tumor environment. Tumor sections derived from Protocol 3 are immunostained for fibronectin and smooth muscle actin, and the orientation of the fibers and cell shape are quantified. Fibronectin fiber orientation is further correlated with the amount of metastasis. It is a replication of experiments reported in Figure 7Cd and Supplemental Figure S7Cc.

#### Sampling

■ Experiment has 3 cohorts:
  ○ Cohort 1: LM-4175 cells alone.
  ○ Cohort 2: LM-4175 cells co-injected with Cav1 WT pMEFs.
  ○ Cohort 3: LM-4175 cells co-injected with Cav1 KO pMEFs.
■ Stained for:
  ○ Fibronectin.
  ○ SMA.
  ○ Control staining:
    - Isotype control.
    - Secondary antibody only control.
■ Experiment will use the following number of primary tumors with 2 sections stained per tumor, with additional sections for controls, and 5 regions imaged per section for a minimum power of 80%:
  ○ See power calculations section for details.
    - Cohort 1: 4 tumors.
    - Cohort 2: 7 tumors.
    - Cohort 2: 7 tumors.
■ Experiment will analyze at least the following number of SMA + cells from the following cohorts for a minimum power of 80%:
  ○ The original lab collected an average of 10–20 cells/region.
  ○ The replication will collect 5 regions/tumor.
  ○ See Power calculations section for details.
    - Cohort 1: 85 images.
    - Cohort 2: 85 images.
    - Cohort 3: 65 images.

#### Materials and reagents

**Table:** 

Reagent	Type	Manufacturer	Catalog #	Comments
Fluoroshield	Chemical	Sigma–Aldrich	F6182	Included during communication with original authors. Original lab used Permafluor.
Acetone	Chemical	Specific brand information will be left up to the discretion of the replicating lab and recorded later		
Chloroform	Chemical			
PBS, without MgCl_2_ and CaCl_2_	Buffer	Sigma–Aldrich	D8537	Originally not specified
Bovine serum albumin	Chemical	Sigma–Aldrich	A9647	Originally not specified
Rabbit IgG isotype control	Antibodies	Sigma–Aldrich	I5006	Originally not specified
Rabbit anti-fibronectin	Antibodies	Sigma–Aldrich	F3648	Stock = 0.5–0.7 mg/ml; Dilute 1:200
Mouse IgG2a isotype control	Antibodies	Sigma–Aldrich	M5409	Originally not specified
Mouse anti-α-smooth muscle actin	Antibodies	Sigma–Aldrich	A5228	Stock = ∼2 mg/ml; Dilute 1:100
Alexa 594 conjugated donkey anti-rabbit IgG	Antibodies	Jackson Immuno Research	711-545-152	Original lab used goat-anti-rabbit-Cy3
Alexa 647 conjugated donkey anti-mouse IgG	Antibodies	Jackson Immuno Research	715-605-151	Originally not specified
Hoechst stain 33,258	Stain	Sigma–Aldrich	14,530	Dilute 1:1000
Confocal microscope	Instrument	Zeiss	LSM510	Original was Leica SPE (communication with original authors)
Image acquisition software	Software	Zeiss	ZEN 2009	Originally not specified
Metamorph microscopy automation and imaging analysis software	Software	Molecular Devices	6.2r1	
Excel	Software	Microsoft		

#### Procedure

1. Cut primary tumor from Protocol 3 on a microtome with a section thickness of 8 µm and mount on slides.
  a. Cut at least 2 sections from each tumor.
2. Fix and permeabilize sections:
  a. Place sections in −20°C acetone and incubate at −20°C for 5 min.
  b. Place sections in −20°C acetone:chloroform (1:1) solution and incubate at −20°C for 5 min.
  c. Place sections in −20°C acetone and incubate at −20°C for 5 min.
  d. Wash sections 2 times in 1× PBS.
3. Incubate sections in 1× PBS supplemented with 2% BSA for 20 min at room temperature. Wash 2 times in 1× PBS.
4. Incubate sections in both primary antibodies diluted in 1× PBS supplemented with 2% BSA overnight at 4°C. Include additional sections for controls.
  a. Rabbit-anti-fibronectin; use at 1:200 dilution.
  b. Mouse-anti-αSMA; use at 1:100 dilution.
  c. Control staining conditions:
    i. Isotype control; use at same concentration as primary.
    ii. secondary antibody only controls.
5. Wash 2 times in 1× PBS.
6. Incubate sections in secondary antibody and Hoechst dye diluted in 1× PBS supplemented with 2% BSA for 1 hr at 37°C.
  a. Alexa 594 donkey-anti-rabbit; use at manufacturer's recommended dilution.
  b. Alexa 647 donkey-anti-mouse; use at manufacturer's recommended dilution.
  c. Hoechst dye; use at 1:1000 dilution.
7. Randomly image 5 independent regions per section at 20× magnification using a confocal microscope.
  Note: if sections are unable to be imaged due to necrosis, autofluorescence, low fibronectin deposition, or damage during the staining procedure, take images, and exclude from analysis with indicated reason.
  a. Total number of regions per tumor is 5 (not including control images).
  b. Acquire z-stack for each region with 0.5 µm-thick z-slices.
  c. Use a 20× objective with a minimum numerical aperture (NA) of 0.70.
8. Blindly quantify intratumoral orientation of fibronectin fibers and SMA + cells and elliptical form factor of SMA + cells. For details see Amatangelo and colleagues (Amatangelo et al., 2005).
  Note: if images are unable to be evaluated due to necrosis, autofluorescence, low fibronectin deposition, or damage during the staining procedure, record as such.
  a. Overlay z-slices to make reconstituted views of the corresponding 3-D fibers for each region.
  b. Subject reconstituted 3-D projections to identical modification and digital filters as described below using MetaMorph offline 6.2r1 imaging analysis software.
  c. Reduce non-specific background with the Flatten Background function by selectively darkening objects with a pixel area greater than 15.
  d. Create a binary image by selecting a 35% threshold at the maximum internal intensity option using the internal threshold function.
  e. Count all fibers recognized, as well as their orientation (angle relative to x-axis) using the integrated morphometry analysis function. Use auto-threshold for light objects and measure angle of displayed objects.
  f. Approximate relative angles to the nearest 10th degree using the rounding function on Microsoft's Excel software and determine the mode angle for each image. Arbitrarily set the mode angle to 0° for each image.
  g. Quantify the elliptical form factor (EF = length/breadth) of SMA + cells using the integrated morphometry analysis function.

#### Deliverables

■ Data to be collected:
  ○ Image stacks of fibronectin, SMA, and Hoescht-stained tumor sections for all conditions, including controls and any images taken, but unable to be analyzed (compare to Figure 7Cc and S7Cb).
  ○ Raw data and summary data of fibronectin fiber orientation (compare to Figure S7Cc).
  ○ Raw data and summary data of elliptical form factor for SMA + cells (compare to Figure S7Cc).
  ○ Raw data and summary data of SMA + cells fiber orientation (compare to Figure S7Cc).

#### Confirmatory analysis plan

This replication attempt will perform the following statistical analysis listed below:

■ Statistical analysis:
  ○ Wilcoxon-Mann Whitney test of percent of fibronectin fibers within ± 20° per tumor by for the following comparisons with the Bonferroni correction:
    Note: in order to enable a direct comparison to how the original data was analyzed, uncorrected tests will also be performed.
    1. LM-4175 only to LM-4175 co-injected with Cav1 WT.
    2. LM-4175 co-injected with Cav1 WT to LM-4175 co-injected with Cav1 KO.
  ○ Wilcoxon–Mann Whitney test of elliptical form factor per SMA + cells for the following comparisons with the Bonferroni correction:
    1. LM-4175 only to LM-4175 co-injected with Cav1 WT.
    2. LM-4175 co-injected with Cav1 WT to LM-4175 co-injected with Cav1 KO.
  ○ Wilcoxon–Mann Whitney test of percent of SMA + cells fibers within ± 20° per tumor by for the following comparisons with the Bonferroni correction:
    1. LM-4175 only to LM-4175 co-injected with Cav1 WT.
    2. LM-4175 co-injected with Cav1 WT to LM-4175 co-injected with Cav1 KO.
  ○ Spearman's rho correlation of intratumoral fibronectin fibers within ± 20° vs number of total metastasis per mouse (cohorts combined).
■ Meta-analysis of effect sizes:
  ○ Compute the effect sizes of each comparison, compare them against the reported effect size in the original paper and use a random effects meta-analytic approach to combine the original and replication effects, which will be presented as a forest plot.

#### Known differences from the original study

The replication attempt is not including the experimental condition of p190RhoGAP knockdown in Cav1 KO pMEFs. All known differences, if any, are listed in the materials and reagents section above, indicated by an asterisk, with the originally used item listed in the comments section. The comments section also lists if the source of original item was not specified. All differences have the same capabilities as the original and are not expected to alter the experimental design.

#### Provisions for quality control

Isotype and secondary antibody only controls will be included. If a section or image is unable to be quantified, due to necrotic damage, autofluroescence, low fibronectin deposition, or damage during the staining procedure, this data will be excluded from the analysis, similar to the original study, but recorded. The objective used to acquire the original images, that were subsequently used for the analysis was a Leica HCX PL APO CS 20×/0.70 IMM UV (communication with original authors). The replication will use a 20× objective with a minimum NA of 0.70. Images will be blindly and randomly taken and evaluated, and all of the raw data, including the control images and analysis files, will be uploaded to the project page on the OSF (https://osf.io/7yqmp) and made publically available.

## Power calculations

For additional details on power calculations, please see analysis scripts and associated files on the Open Science Framework:■ https://osf.io/q3e4u/.

### Protocols 1 and 2

Not applicable.

### Protocol 3

#### Total metastatic foci per mouse

Summary of original data (provided by original authors).

**Table:** 

Dataset being analyzed	Mean	SD	N
LM-4175 only	2.50	1.975	6
LM-4175 + Cav1 WT pMEFs	28.42	22.24	12
LM-4175 + Cav1 KO pMEFs	11.67	11.10	15

Power calculations performed with G*Power software, version 3.1.7. (Faul et al., 2007).

##### Test family

■ *t* test: means: Wilcoxon–Mann–Whitney test (two groups, one tail), alpha error = 0.0167.

**Table:** 

Group 1	Group 2	Pooled SD	Effect size d	A priori power	Group 1 sample size	Group 2 sample size
LM-4175 only	LM-4175 + Cav1 WT pMEFs	18.47	1.403155	80.7%	7	21
LM-4174 only	LM-4175 + Cav1 KO pMEFs	9.58	1.390827*	80.0%*	7	21
LM-4175 + Cav1 WT pMEFs	LM-4175 + Cav1 KO pMEFs	16.93	0.989463	81.9%	21	21

* A sensitivity calculation was performed since the original data showed a non-significant effect. This is the effect size that can be detected with 80% power.

#### Primary tumor growth

Summary of original data (provided by original authors).

**Table:** 

Dataset being analyzed	Mean	SD	N
LM-4175 only	2.337 × 10^10^	1.856 × 10^10^	6
LM-4175 + Cav1 WT pMEFs	2.825 × 10^10^	3.901 × 10^10^	13
LM-4175 + Cav1 KO pMEFs	2.312 × 10^10^	1.368 × 10^10^	15

Analysis of original data: (Kruskal–Wallis; performed with GraphPad Prism, version 6.0).

**Table:** 

Kruskal–Wallis statistic	p-value
0.8878	0.6415

Power calculations performed with G*Power software, version 3.1.7. (Faul et al., 2007).

##### Test family

■ F test: ANOVA: Fixed effects, omnibus, one-way, alpha error = 0.05.

**Table:** 

Groups	Effect size *f*	A priori power	Total sample size
LM-4175 only, LM-4175 + Cav1 WT pMEFs, LM-4175 + Cav1 KO pMEFs	0.504525*	80.0%*	41† (3 groups)

* A sensitivity calculation was performed since the original data showed a non-significant effect. This is the effect size that can be detected with the sample size reported and 80% power.

† Since the non-parametric Kruskal–Wallis test will be performed for the analysis instead of an ANOVA, the sensitivity calculation was performed with a ∼15% adjustment in sample size to calculate the effect size that can be detected with 80% power. The total sample size of 49, which comes from the total metastatic foci per mouse sample size calculation, was reduced by ∼15%–41 for this calculation to estimate the detectable effect size.

### Protocol 4

#### Percent of fibronectin fibers within ± 20°

Summary of original data (obtained from Figure S7Cc).

**Table:** 

Dataset being analyzed	N	Mean	SEM	SD
LM-4175 only	5	36.8	0.7	1.565
LM-4175 + Cav1 WT pMEFs	8	50.3	2.3	6.505
LM-4175 + Cav1 KO pMEFs	10	41.5	1.1	3.479

Power calculations performed with G*Power software, version 3.1.7. (Faul et al., 2007).

##### Test family

■ *t* test: Means: Wilcoxon–Mann–Whitney test (two groups, one tail), alpha error = 0.025.

**Table:** 

Group 1	Group 2	Pooled SD	Effect size d	A priori power	Group 1 sample size	Group 2 sample size
LM-4175 only	LM-4175 + Cav1 WT pMEFs	5.27	2.559575	82.7%*	4	4*
LM-4175 + Cav1 WT pMEFs	LM-4175 + Cav1 KO pMEFs	5.03	1.748808	83.1%	7	7

* 7 tumors will be used based on the WT vs KO comparison making the power 94.0%.

#### Elliptical form factor of SMA + cells

Summary of original data (obtained from Figure S7Cc).

**Table:** 

Dataset being analyzed	N	Mean	SEM	SD
LM-4175 only	224	1.70	0.03	0.449
LM-4175 + Cav1 WT pMEFs	1246	2.14	0.03	1.059
LM-4175 + Cav1 KO pMEFs	763	1.68	0.02	0.5524

Power calculations performed with G*Power software, version 3.1.7. (Faul et al., 2007).

##### Test family

■ *t* test: Means: Wilcoxon–Mann–Whitney test (two groups, one tail), alpha error = 0.025.

**Table:** 

Group 1	Group 2	Pooled SD	Effect size d	A priori power	Group 1 sample size	Group 2 sample size
LM-4175 only	LM-4175 + Cav1 WT pMEFs	0.99	0.444072	80.3%	85	85
LM-4175 + Cav1 WT pMEFs	LM-4175 + Cav1 KO pMEFs	0.90	0.510625	80.6%	65	65

#### Percent of SMA + fibers within ± 20°

Summary of original data (obtained from Figure S7Cc).

**Table:** 

Dataset being analyzed	N	Mean	SEM	SD
LM-4175 only	5	33.1	4	8.944
LM-4175 + Cav1 WT pMEFs	8	51.3	2	5.657
LM-4175 + Cav1 KO pMEFs	10	35.3	2	6.325

Power calculations performed with G*Power software, version 3.1.7. (Faul et al., 2007).

##### Test family

■ *t* test: means: Wilcoxon–Mann–Whitney test (two groups, one tail), alpha error = 0.025.

**Table:** 

Group 1	Group 2	Pooled SD	Effect size d	A priori power	Group 1 sample size	Group 2 sample size
LM-4175 only	LM-4175 + Cav1 WT pMEFs	7.03	2.588043	83.5%*	4	4*
LM-4175 + Cav1 WT pMEFs	LM-4175 + Cav1 KO pMEFs	6.04	2.648198	85.0%†	4†	4†

* 7 tumors will be used based on the fibronectin fiber orientation analysis making the power 94.4%.

† 7 tumors will be used based on the fibronectin fiber orientation analysis making the power 99.3%.

#### Correlation of percent of fibronectin fibers within ± 20° and number of metastasis

Original data (obtained from original authors).

The shared data contained 27 XY pairs with a calculated Spearman rho of 0.7488, which is missing 3 pairs included in the published analysis, with a Spearman rho of 0.81. The power calculations were performed on the shared data and Spearman rho, which will give a more conservative sample size to detect the published value.

Power calculations performed with R software, version 3.1.0 (R Development Core Team, 2014).

##### Test family

■ Correlation: Spearman's rho test (one sided), alpha error = 0.05.

**Table:** 

Groups	Number of simulations	A priori power	Total sample size
% fibronectin fibers within ± 20° and number of metastasis	10,000*	83.1%	10

* The shared data from XY pairs was randomly sampled from, with replacement, to create simulated data sets with preserved correlated structure. For a given *n* (the number of observations) 10,000 simulations were run and Spearman's rho was calculated for each simulated data set. The power was then calculated by counting the number of times p ≤ 0.05 and dividing by 10,000.

## References

[bib1] Amatangelo MD, Bassi DE, Klein-Szanto AJ, Cukierman E (2005). Stroma-derived three-dimensional matrices are necessary and sufficient to promote desmoplastic differentiation of normal fibroblasts. The American Journal of Pathology.

[bib2] Capozza F, Trimmer C, Castello-Cros R, Katiyar S, Whitaker-Menezes D, Follenzi A, Crosariol M, Llaverias G, Sotgia F, Pestell RG, Lisanti MP (2012). Genetic ablation of Cav1 differentially affects melanoma tumor growth and metastasis in mice: role of Cav1 in Shh heterotypic signaling and transendothelial migration. Cancer Research.

[bib3] Cerezo A, Guadamillas MC, Goetz JG, Sanchez-Perales S, Klein E, Assoian RK, del Pozo MA (2009). The absence of caveolin-1 increases proliferation and anchorage- independent growth by a Rac-dependent, Erk-independent mechanism. Molecular and Cellular Biology.

[bib4] Chang SH, Feng D, Nagy JA, Sciuto TE, Dvorak AM, Dvorak HF (2009). Vascular permeability and pathological angiogenesis in caveolin-1-null mice. The American Journal of Pathology.

[bib5] Cirri P, Chiarugi P (2011). Cancer associated fibroblasts: the dark side of the coin. American Journal of Cancer Research.

[bib5a] Errington TM, Iorns E, Gunn W, Tan FE, Lomax J, Nosek BA (2014). An open investigation of the reproducibility of cancer biology research. eLife.

[bib6] Faul F, Erdfelder E, Lang AG, Buchner A (2007). G*Power 3: a flexible statistical power analysis program for the social, behavioral, and biomedical sciences. Behavior Research Methods.

[bib7] Goetz JG, Lajoie P, Wiseman SM, Nabi IR (2008). Caveolin-1 in tumor progression: the good, the bad and the ugly. Cancer Metastasis Reviews.

[bib8] Goetz JG, Minguet S, Navarro-Lerida I, Lazcano JJ, Samaniego R, Calvo E, Tello M, Osteso-Ibanez T, Pellinen T, Echarri A, Cerezo A, Klein-Szanto AJ, Garcia R, Keely PJ, Sanchez-Mateos P, Cukierman E, Del Pozo MA (2011). Biomechanical remodeling of the microenvironment by stromal caveolin-1 favors tumor invasion and metastasis. Cell.

[bib9] Kalluri R, Zeisberg M (2006). Fibroblasts in cancer. Nature Reviews. Cancer.

[bib10] Kamijo T, Zindy F, Roussel MF, Quelle DE, Downing JR, Ashmun RA, Grosveld G, Sherr CJ (1997). Tumor suppression at the mouse INK4a locus mediated by the alternative reading frame product p19ARF. Cell.

[bib11] Linke SP, Krajewski S, Bremer TM, Man AK, Zeps N, Spalding L (2010). Abstract P3-10-42: stromal caveolin-1 is a powerful marker that further enhances a multi-marker prognostic profile. Cancer Research.

[bib12] Ma X, Liu L, Nie W, Li Y, Zhang B, Zhang J, Zhou R (2013). Prognostic role of caveolin in breast cancer: a meta-analysis. The Breast.

[bib13] Minn AJ, Gupta GP, Siegel PM, Bos PD, Shu W, Giri DD, Viale A, Olshen AB, Gerald WL, Massague J (2005). Genes that mediate breast cancer metastasis to lung. Nature.

[bib14] Parton RG, del Pozo MA (2013). Caveolae as plasma membrane sensors, protectors and organizers. Nature Reviews. Molecular Cell Biology.

[bib15] Provenzano PP, Eliceiri KW, Campbell JM, Inman DR, White JG, Keely PJ (2006). Collagen reorganization at the tumor-stromal interface facilitates local invasion. BMC Medicine.

[bib16] Razani B, Engelman JA, Wang XB, Schubert W, Zhang XL, Marks CB, Macaluso F, Russell RG, Li M, Pestell RG, Di Vizio D, Hou H, Kneitz B, Lagaud G, Christ GJ, Edelmann W, Lisanti MP (2001). Caveolin-1 null mice are viable but show evidence of hyperproliferative and vascular abnormalities. The Journal of Biological Chemistry.

[bib17] Ren M, Liu F, Zhu Y, Li Y, Lang R, Fan Y, Gu F, Zhang X, Fu L (2014). Absence of caveolin-1 expression in carcinoma-associated fibroblasts of invasive micropapillary carcinoma of the breast predicts poor patient outcome. Virchows Archiv.

[bib18] Righi L, Cavallo MC, Gatti G, Monica V, Rapa I, Busso S, Albera C, Volante M, Scagliotti GV, Papotti M (2014). Tumor/stromal caveolin-1 expression patterns in pleural mesothelioma define a subgroup of the epithelial histotype with poorer prognosis. American Journal of Clinical Pathology.

[bib19] Shatz M, Liscovitch M (2008). Caveolin-1: a tumor-promoting role in human cancer. International Journal of Radiation Biology.

[bib20] Simpkins SA, Hanby AM, Holliday DL, Speirs V (2012). Clinical and functional significance of loss of caveolin-1 expression in breast cancer-associated fibroblasts. The Journal of Pathology.

[bib21] Sotgia F, Martinez-Outschoorn UE, Howell A, Pestell RG, Pavlides S, Lisanti MP (2012). Caveolin-1 and cancer metabolism in the tumor microenvironment: markers, models, and mechanisms. Annual Review of Pathology.

[bib22] R Development Core Team (2014). R: a language and environment for statistical computing. R Foundation for Statistical Computing.

[bib23] Yang G, Timme TL, Naruishi K, Fujita T, Fattah el MA, Cao G, Rajagopalan K, Troung LD, Thompson TC (2008). Mice with cav-1 gene disruption have benign stromal lesions and compromised epithelial differentiation. Experimental and Molecular Pathology.

[bib24] Zhao X, He Y, Gao J, Fan L, Li Z, Yang G, Chen H (2013). Caveolin-1 expression level in cancer associated fibroblasts predicts outcome in gastric cancer. PLOS ONE.

