# Supplementary material for: Registered report: Biomechanical remodeling of the microenvironment by stromal caveolin-1 favors tumor invasion and metastasis
Source: eLife. 2015 Jul 16;4:e04796. doi: 10.7554/eLife.04796 (PMC4503935; doi:10.7554/eLife.04796)
Supplement: Related texts

# Article commentary: Decision letter

**DOI:** 10.7554/eLife.04796.002

**Affiliations:** University College London, United Kingdom

eLife posts the editorial decision letter and author response on a selection of the published articles (subject to the approval of the authors). An edited version of the letter sent to the authors after peer review is shown, indicating the substantive concerns or comments; minor concerns are not usually shown. Reviewers have the opportunity to discuss the decision before the letter is sent (see review process). Similarly, the author response typically shows only responses to the major concerns raised by the reviewers.

Thank you for sending your work entitled “Registered report: Biomechanical remodeling of the microenvironment by stromal Cav1 favors tumor invasion and metastasis” for consideration at *eLife*. Your article has been evaluated by Fiona Watt (Senior editor), a Reviewing editor, and four reviewers, one of whom has direct statistical expertise.

The following individuals responsible for the peer review of your submission have agreed to reveal their identity: Miguel Del Pozo (Reviewer 1), Peter Friedl (Reviewer 2), and Dawn Teare (Reviewer 4). Reviewer 3 remains anonymous.

The Reviewing editor and the reviewers discussed their comments before we reached this decision, and the Reviewing editor has assembled the following comments to help you prepare a revised submission.

Overall, the reviewers agree on the choice of the key experiments and the experimental approach. However, a number of points have been raised that need to be addressed in a revised Registered Report before proceeding with the experiments:

The authors should provide a more extensive and more balanced discussion of the literature on the function of caveolin-1 in different cancers; some missed references are listed in the comments of reviewer 1 below. Importantly, the Capozza et al. paper, presented as in contradiction with the findings by Goetz et al., may not be in contradiction since Capozza et al. focused on the size of the primary tumours whereas Goetz et al. focused on metastasis (see also comments from Reviewer 1 below for more details). This should be clarified. If the authors wish to insist on a discrepancy between the Capozza et al. and the Goetz et al. studies, it would be a good idea to inject the B16F10 cells used by Capozza et al. (Reviewer 3's suggestion), to clarify whether the discrepancy may be caused by a difference in cell lines.

There are a number of experimental points listed in the reviews comments appended below that need to be clarified.

Extracts from Reviewer #1:

A significant overstatement made by the authors of this Reproducibility Project pertains to protocol 2. They claim that there is a contradiction between the tested paper (Goetz et al. Cell 2011) and another one by the Lisanti group: “In contrast to these findings, Capozzo and colleagues showed that intradermal coinjection of nude mice with B16F10 melanoma cells and Cav1 KO neonatal dermal fibroblasts increased primary tumor growth when compared to coinjection of tumor cells with WT fibroblasts (Capozza et al., 2012).” We disagree that there is conflict here, since the original paper by Goetz et al. was focused on studying changes in stromal remodeling and, as a consequence of them, correlative changes in tumor local invasion and distant metastasis. The growth of the primary tumor was not the main focus of Goetz et al., and hence it was not as critically evaluated as ECM remodeling or metastasis, as it was the case for Capozza et al. Due to the nature of the allograft/xenograft experiments of Figure 7, focused on evaluating ECM remodeling and the appearance of metastasis, it was also possible to measure the size of the primary tumor, but this was not as critically monitored as the metastasis formation. Hence, we believe that it is not correct to claim that one of the main conclusions of the original paper is that the growth of the primary tumor is not affected by the absence of Cav1 expression in fibroblasts. Therefore, we believe is not correct to include primary tumor growth as one of the statistical analysis to be performed in protocol 2. In the experimental approach that Fiering et al. have chosen, the primary tumor growth was not significantly different between any of the studied conditions. However, primary tumor growth was decreased in the absence of Cav1 expression in mammary gland allografts (Figure 7A and S7A) and in mammary gland xenografts (Figure 7B and S7B). It is important to note that in these approaches, the whole mammary gland was deficient for Cav1 expression, not only the fibroblasts, and hence they are not exactly comparable. In the same regard, Capozza et al. used neonatal dermal fibroblasts, which are not exactly the same as MEFs. Further, Fiering et al. fail to cite other papers that apparently contradict Capozza et al. and are in line with results of Figure S7A and S7B. One example comes from the Dvorak group, which showed that subcutaneous injection of B16 melanoma cells in Cav1 KO mice leads to reduced tumor growth compared to injection of tumor cells in WT mice (Chang et al., Am J Path 2009). Fiering et al. should also cite this work, but, even more importantly, these differences highlight the complexity of this biological problem, and the potential difficulty to reproduce observations made in several labs.

In the particular case of the subcutaneous xenograft model (Figure 7C), the main conclusions to be reproduced should thus be:

1) Absence of Cav1 expression in fibroblasts inhibits metastasis of LM-4175 breast tumor cells.

2) Absence of Cav1 expression in fibroblasts results in a less anisotropic ECM (with a lower proportion of parallel Fibronectin fibers).

3) A statistical significant correlation between the anisotropy of the intratumoral fibers and the total number of metastasis per mouse (Spearman's rho correlation).

4) Presence or absence of Cav1 expression in fibroblast modifies cell shape (elliptical factor of SMA+ cells).

Do the authors accurately summarize the literature, especially with respect to other direct replications? No, and this is a critical issue that we have observed in reviewing the manuscript by Fiering et al. We do not agree with the selection of papers made to introduce the subject of Caveolin-1 expression in tumor stroma. The papers chosen belong mostly to the same group, which consistently reports Caveolin-1 as a tumor suppressor. However, caveolin-1 expression in cancer is a very controversial subject of research. There are thousands of papers and one can virtually find papers showing both increased and decreased expression in any particular type of cancer (Parton and del Pozo, Nature Reviews Mol Cell Biol 2013). A review that carefully evaluated caveolin-1 expression in multiple types of cancers from primary to metastatic stages, concluded a general trend in which Caveolin-1 appears to act as a tumor suppressor at early stages of cancer progression, but it is up-regulated in several multidrug-resistant and metastatic cancer cell lines and human tumor specimens, positively correlating with tumor stage and grade in numerous cancer types (Shatz and Liscovitch, Int J Radiat Biol 2008). One of the conclusions of the original article by Goetz et al. was that part of this variability could stem from Cav1 expression in stromal rather than tumor cells. In fact, the papers cited in the introduction plus some others are already confirming this.

Therefore, a more thorough review of the existing literature should be performed in order to better present the complexity of this particular aspect. For example, loss of Cav1 function in stromal cells of various organs leads to benign stromal lesions responsible for abnormal growth and differentiation of the epithelium and to dramatic reductions in life span (Yang et al., Exp Mol Path 2008). Consistently, Goetz et al. showed that Cav1 expressed in fibroblasts can modulate normal cell morphology via force-dependent ECM remodelling. Thus, reports like Yang et al. showing that lack of stromal Cav1 could disturb normal tissue architecture should also be cited by Fiering et al.

Another important point is that, in general, the papers cited in the Introduction do not use specific markers of specific stromal types, so it is not possible to be certain whether they are CAFs or some other stromal cell type. Goetz et al. used several fibroblast markers throughout the study, and in particular Cav-1/α-SMA colocalization in the human cancer samples. These differences should be outlined by Fiering et al.

To the best of our knowledge, no direct replications of the original study have been reported. However, several recent studies (not included by the authors) have addressed the role of stromal Cav1 expression in different types of tumors. Righi et al. have recently published that high Cav1-expression in the stroma is associated with a worse patient outcome in malignant pleural mesothelioma (Righi et al., Am J Clin Pathol 2014), although the molecular mechanism behind this conclusion was not experimentally addressed. These results are in line with the data of the original article on a breast cancer tissue microarray analysis (Figure 5C) showing that increased Cav1 in the stroma correlated with decreased survival. Moreover, Linke et al. have reported that stromal Cav1 (often surrounding “nests” of tumor cells) was a powerful univariate prognostic marker that remained significant in the context of a pre-existing multi-marker Breast Cancer diagnostic commercial profile (Linke SP, Krajewski S, Bremer TM, Man AK, Zeps N, Spalding L. “Stromal caveolin-1 is a powerful marker that further enhances a multi-marker prognostic profile”. Cancer Res. 2010;70(24 Suppl):Abstract P3-10-42). Further, results presented in 2014 ASCO Annual Meeting by the Spanish Breast Cancer Research Group (GEICAM) confirm positive stromal CAV-1 as a prognostic breast cancer marker.

In contrast, another study focused of gastric cancer reported that low stromal-Cav1 expression was associated with worse patient outcome (Zhao et al., PLoS One 2013). The molecular mechanism was not addressed and the differences with previously published results (including the original study) poorly discussed, but this already shows the tremendous variability existing in this complex subject.

As mentioned in the previous section, when referring to the Capozza et al. paper the authors should also cite the work of the contrasting paper by the Dvorak group (Chang et al., Am J Path 2009). As in the other examples mentioned throughout this section, it is not clear why Fiering et al. only cite those papers in apparent contrast with the original article.

Therefore, the authors of this Reproducibility Project should undertake a comprehensive review of the literature on this subject. In the current version they have only chosen those papers that somehow contradict the original one, but they do not mention to what extent they are different (type of tumor, TMA vs. mouse experiments, etc.) and missed important literature that should be included, some of which is consistent with Goetz et al.

Are the proposed experiments appropriately designed? The proposed experiment is appropriately designed, although several small modifications should be highlighted:

1) Protocol 1, procedure points 5-6 do not reflect the original protocol and should be indicated by an asterisk.

2) Protocol 2, it must clearly indicate that the MEFs co-injected are primary MEFs.

3) Protocol 2, procedure point 3b. Nude mice do not require shaving since they do not have any hair; shaving will thus induce unnecessary skin irritation, which could lead to stromal activation.

4) Protocol 2, procedure point 3d. It is important not to take the needle out of the skin till the matrigel has gelified. Gelification takes very short time at body temperature (around a minute).

5) Protocol 2, procedure point 6. Although in the original study the final analysis point was set at 70 days, it is important to keep some kind of flexibility for this end-point. The investigators must consider shortening this time if the number of mice euthanized compromise the success of the whole study (due to the reasons explained in point 5).

6) Protocol 3, sampling. Please stain at least 2 sections per tumor, since some sections are difficult to evaluate due to necrosis, autofluorescence or damage during the staining procedure.

7) Protocol 3, materials. The authors plan to use Alexa488 conjugated donkey anti-rabbit IgG (although later in procedure point 6, they claim to use goat-anti rabbit FITC, please check this inconsistency). In the original study, goat-anti rabbit Cy3 was used. This selection is not trivial, since the plasmid used to express Luciferase in the LM-4175 cells (HSV-tk1-GFP-luc) contains an IRES-GFP sequence. Thus, LM-4175 cells are green (although not really bright), and therefore this fluorescence channel must not be used for additional staining.

8) Protocol 3, materials. The authors plan to use Alexa647 conjugated donkey anti-mouse IgG as we have done. However, later in procedure section, point 6, they claim to use goat-anti mouse CF640R, please check this inconsistency.

Are the proposed statistical analyses rigorous and appropriate? The use of Wilcoxon-Mann-Whitney test to compare total metastatic foci counts in the three groups is well sounded and agrees with the statistical analysis from the original paper; however, since the original paper did report raw p-values only, we believe it would be more appropriate to report also non-corrected p-values in the reviewed paper. On top of that, post-hoc methods based on the control of the FWER (such as Bonferroni) tend to be too conservative compared to methods controlling the FDR, which are the ones we believe should be used by Fiering et al. The use of Bonferroni would significantly dampen the probability to find significant differences, which we believe is somehow unfair for this Reproducibility Project.

On the other hand, Fiering et al. report that they will perform a meta-analysis with the results of the original paper and the new results, but there are no details about the methodology used for such a meta-analysis.

What can the replication team do to maximize the quality of the replication?

Please see the modifications of the protocol highlighted above. Although with 3 regions imaged per sections the authors claim to obtain a minimum power of 80%, some tumors are difficult to image (necrotic damage, autofluorescence, low fibronectin deposition or damage due to preparation or manipulation). For these reasons, we would highly recommend to stain at least two sections per tumor and to image at least five regions per section, although the more the better.

Extracts from Reviewer #2:

Several points not specified in Goetz et al. 2011 should be clarified with the original authors, before initiating the experiments. *eLife* has verified these points with the original authors and the responses follow the questions:

The order code of the nude mice and their age range when used for the study.

Nude mice, official name from Harlan: Athymic Nude Mouse -Hsd: Athymic Nude-Foxn1^nu^.

And their age range when used for the study: 8-10 weeks when they arrive, about 10-12 when experiment was done.

Whether DMEM with high glucose was used for in vitro culture of MEFs. Normal DMEM, not high glucose.

Original matrigel stock concentration, so the used relative dilution yields the same final concentration for implantation.

Typical protein concentrations for BD Matrigel Matrix are between 9-12 mg/ml.

Protocol 2:

For metastasis detection, a range of exposure times from 20s to 2 min was used to detect both large and small metastases. Thus, each mouse and excised organ should be assessed to reach maximum sensitivity and count a maximum of micrometastases. To reach high sensitivity, when monitoring the lungs, liver and brain, the lower abdomen (containing tumor and luminescence in the bladder) should be shielded during longer exposure times. Also, for metastasis detection of excised organs, 2 min should be included as longest exposure time.

Protocol 3:

Irrespective of the power calculation, because of expectable intra-tumor variation of stromal density and organization, several sections from the same tumor should be analyzed for fibronectin alignment and cell elongation. The analysis of only 3 fields from a single section may be flawed by accidental sample variability and insufficient representativity.

Extracts from Reviewer #4:

For protocol 2 and 3 the exact details of what was found in the original paper are reported (generally groups means and SDs) so I easily can follow this logic of how the sample size/power calculations have been derived. I have a number of questions regarding assumptions made in these sample size calculations:

1) Often the SD used in the power calc is estimated on a group of size between 6 and 15 so these estimates will tend to be underestimates. This is not so much of a problem when the effect sizes are approximately 1 SD but it could still be taken account of. I cannot replicate the pooled SDs used in the tables – mine come out slightly larger.

2) Sometimes the type 1 error is adjusted for multiple testing and sometimes it is not. If the same animals are studied for several outcomes then these should be treated as multiple testing problems. When several organ sites are examined this must surely be the same animals?

3) It seems odd to me that the replication study wants to replicate results that were not statistically significant in the original study? In these cases they argue that they select an effect size that they have 80% power to detect.

4) There are many hypotheses being tested in this replication. Are they all equally important? Are some of them nested? If half of the tests are statistically significant will this be regarded as a validation of the original report? I am not confident that these sample sizes will have sufficient power for so many repeated tests.

5) There is some confusion in statistical language which may be a feature of the G*power program. The sample size calculations state that *t*-tests (to detect standardised effects) will be used but the analysis plan uses non-parametric tests (Kruskall Wallis and Mann Whitney, etc).

[Editors' note: further revisions were requested prior to acceptance, as described below.]

Thank you for resubmitting your work entitled “Registered report: Biomechanical remodeling of the microenvironment by stromal Cav1 favors tumor invasion and metastasis” for further consideration at *eLife*. Your revised article has been favorably evaluated by Fiona Watt (Senior editor), a member of the Board of Reviewing Editors, and three reviewers. The manuscript has been improved but there are a few minor remaining issues that need to be addressed before acceptance, as outlined below:

Both Abstract and Introduction should clearly state, as it was stated in the first version of this manuscript, that the 50 papers being replicated are the top 50 most impactful ones in the cancer biology field, and not just 50 random papers from this field. This critical piece of information has now been removed from both Abstract and Introduction. Also, the Abstract should state clearly that the authors are replicating not the whole paper, but only a fraction of it.

Several important controls are missing: it would be important to check by WB that the pMEFs generated are actually KO for caveolin-1. Also, to make sure that WT pMEFs have increased expression of (at least) smooth muscle actin (SMA) compared to KO pMEFs, as a marker of increased activation and ECM remodeling capabilities of these pMEFs.

If the authors wish to insist on the discrepancy between the Capozza et al. and the Goetz et al. studies, they should inject the B16F10 cells used by Capozza to clarify whether the discrepancy may be caused by a difference in cell lines.

Regarding statistics, the reviewers were confused by the answer of the authors to question 3 of Reviewer #4. If the authors want to see if the effect of these distinct groupings is equivalent, it seems one should not do a superiority power calculation but rather a bioequivalence. The authors have used a 15% adjustment, which is what is done to convert numbers in a parametric calculation to a non-parametric calculation. The answer seems to imply (in protocol 2) that the authors found they needed 39 animals and adjusted this to 48). Then the authors used the sample size to see what effect size this would have 80% power to detect? However, the reviewers did not follow how the authors got to 39 animals in the first place. Could the authors clarify this point?

Finally, the authors should indeed report also the raw *p*-values. This is very important because corrected *p*-values (especially with Bonferroni) will always be less significant than the raw ones, so some of the significant results could be “lost” if only corrected *p*-values were reported.

# Reply: Author response

**DOI:** 10.7554/eLife.04796.003

The authors should provide a more extensive and more balanced discussion of the literature on the function of caveolin-1 in different cancers; some missed references are listed in the comments of reviewer 1 below. Importantly, the Capozza et al. paper, presented as in contradiction with the findings by Goetz et al., may not be in contradiction since Capozza et al. focused on the size of the primary tumours whereas Goetz et al. focused on metastasis (see also comments from Reviewer 1 below for more details). This should be clarified. If the authors wish to insist on a discrepancy between the Capozza et al. and the Goetz et al. studies, it would be a good idea to inject the B16F10 cells used by Capozza et al. (Reviewer 3's suggestion), to clarify whether the discrepancy may be caused by a difference in cell lines.

We have included a more balanced discussion of the literature on caveolin-1, including the references in the comments of Reviewer #1. And we have removed the indicated discrepancy between the Capozza et al. and the Goetz et al. studies and included the results of the Goetz et al., Capozza et al., and Chang et al. studies.

There are a number of experimental points listed in the reviews comments appended below that need to be clarified.

Extracts from Reviewer #1:

*A significant overstatement made by the authors of this Reproducibility Project pertains to protocol 2. They claim that there is a contradiction between the tested paper (Goetz et al.* *Cell 2011) and another one by the Lisanti group:* “*In contrast to these findings, Capozzo and colleagues showed that intradermal coinjection of nude mice with B16F10 melanoma cells and Cav1 KO neonatal dermal fibroblasts increased primary tumor growth when compared to coinjection of tumor cells with WT fibroblasts (**Capozza et al., 2012**).*” *We disagree that there is conflict here, since the original paper by Goetz et al. was focused on studying changes in stromal remodeling and, as a consequence of them, correlative changes in tumor local invasion and distant metastasis. The growth of the primary tumor was not the main focus of Goetz et al., and hence it was not as critically evaluated as ECM remodeling or metastasis, as it was the case for Capozza et al. Due to the nature of the allograft/xenograft experiments of Figure 7, focused on evaluating ECM remodeling and the appearance of metastasis, it was also possible to measure the size of the primary tumor, but this was not as critically monitored as the metastasis formation. Hence, we believe that it is not correct to claim that one of the main conclusions of the original paper is that the growth of the primary tumor is not affected by the absence of Cav1 expression in fibroblasts. Therefore, we believe is not correct to include primary tumor growth as one of the statistical analysis to be performed in protocol 2. In the experimental approach that Fiering et al. have chosen, the primary tumor growth was not significantly different between any of the studied conditions. However, primary tumor growth was decreased in the absence of Cav1 expression in mammary gland allografts (Figure 7A and S7A) and in mammary gland xenografts (Figure 7B and S7B). It is important to note that in these approaches, the whole mammary gland was deficient for Cav1 expression, not only the fibroblasts, and hence they are not exactly comparable. In the same regard, Capozza et al. used neonatal dermal fibroblasts, which are not exactly the same as MEFs. Further, Fiering et al. fail to cite other papers that apparently contradict Capozza et al. and are in line with results of* *Figure S7A and S7B. One example comes from the Dvorak group, which showed that subcutaneous injection of B16 melanoma cells in Cav1 KO mice leads to reduced tumor growth compared to injection of tumor cells in WT mice (Chang et al., Am J Path 2009). Fiering et al. should also cite this work, but, even more importantly, these differences highlight the complexity of this biological problem, and the potential difficulty to reproduce observations made in several labs.*

In the particular case of the subcutaneous xenograft model (Figure 7C), the main conclusions to be reproduced should thus be:

1) Absence of Cav1 expression in fibroblasts inhibits metastasis of LM-4175 breast tumor cells.

2) Absence of Cav1 expression in fibroblasts results in an less anisotropic ECM (with a lower proportion of parallel Fibronectin fibers).

3) A statistical significant correlation between the anisotropy of the intratumoral fibers and the total number of metastasis per mouse (Spearman's rho correlation).

4) Presence or absence of Cav1 expression in fibroblast modifies cell shape (elliptical factor of SMA+ cells).

We have included a more balanced discussion of the literature on caveolin-1. And we have removed the indicated discrepancy between the Capozza et al. and the Goetz et al. studies and included the results of the Goetz et al., Capozza et al., and Chang et al. studies as suggested. We also try to focus on the experiments being replicated and any direct replications of them. We agree that the metastasis formation in this model is the primary focus of Protocol 2 and have determined the appropriate sample size to have adequate power for this analysis. As an added aspect of the experimental approach, we have included the primary tumor growth data analysis, just like the original study, with the effect size we would be able to detect, if there is one, with the sample size we are using. The inclusion of this data is also only in respect to this exact model, not to the potential of Cav1 on primary tumor growth in other models, including the ones presented in Goetz et al. (Figures S7A-B). As such, we will restrict our analysis to the experiments being replicated and will not include discussion of experiments not being replicated in this study.

[…] Therefore, the authors of this Reproducibility Project should undertake a comprehensive review of the literature on this subject. In the current version they have only chosen those papers that somehow contradict the original one, but they do not mention to what extent they are different (type of tumor, TMA vs. mouse experiments, etc.) and missed important literature that should be included, some of which is consistent with Goetz et al.

We have included a more balanced discussion of the literature on caveolin-1. Additionally, we have removed the indicated discrepancy between the Capozza et al. and the Goetz et al. studies and included the results of the Goetz et al., Capozza et al., and Chang et al. studies as suggested as well as include the other suggested references. We try to focus on the experiments being replicated and any direct replications of them, instead of a comprehensive review of all the literature on this subject, which includes many conceptual or related experiments, which would be more appropriate for a review.

Are the proposed experiments appropriately designed? The proposed experiment is appropriately designed, although several small modifications should be highlighted:

1) Protocol 1, procedure points 5-6 do not reflect the original protocol and should be indicated by an asterisk.

We have reached out the original authors to clarify the exact protocol that was used. The dissociation of the pMEFs by trypsin was performed in the original study, but we have revised the other steps to reflect the exact protocol used.

2) Protocol 2, it must clearly indicate that the MEFs co-injected are primary MEFs.

We have made this clearer and corrected any references to MEFs as primary MEFs.

3) Protocol 2, procedure point 3b. Nude mice do not require shaving since they do not have any hair; shaving will thus induce unnecessary skin irritation, which could lead to stromal activation.

Thank you for catching this. We have removed this from the procedure.

4) Protocol 2, procedure point 3d. It is important not to take the needle out of the skin till the matrigel has gelified. Gelification takes very short time at body temperature (around a minute).

Thank you for the additional details. We have added this into the procedure.

5) Protocol 2, procedure point 6. Although in the original study the final analysis point was set at 70 days, it is important to keep some kind of flexibility for this end-point. The investigators must consider shortening this time if the number of mice euthanized compromise the success of the whole study (due to the reasons explained in point 5).

Thank you for this information. We have included a statement reflecting the potential of shorting the study time to ensure enough mice are obtained for analysis. If the time is changed because of an increase in having to euthanize mice due to complications, the change in timeframe will be recorded.

6) Protocol 3, sampling. Please stain at least 2 sections per tumor, since some sections are difficult to evaluate due to necrosis, autofluorescence or damage during the staining procedure.

We have updated the protocol to reflect 2 sections per tumor. We have also included the exclusion criteria outlined here that might prevent a section or image from being included in the analysis.

7) Protocol 3, materials. The authors plan to use Alexa488 conjugated donkey anti-rabbit IgG (although later in procedure point 6, they claim to use goat-anti rabbit FITC, please check this inconsistency). In the original study, goat-anti rabbit Cy3 was used. This selection is not trivial, since the plasmid used to express Luciferase in the LM-4175 cells (HSV-tk1-GFP-luc) contains an IRES-GFP sequence. Thus, LM-4175 cells are green (although not really bright), and therefore this fluorescence channel must not be used for additional staining.

Thank you for catching this. We have changed the Alexa488 to Alexa594 so the imaging is not in the same channel as the GFP from the integrated luciferase vector in the LM-4175 cells.

8) Protocol 3, materials. The authors plan to use Alexa647 conjugated donkey anti-mouse IgG as we have done. However, later in procedure section, point 6, they claim to use goat-anti mouse CF640R, please check this inconsistency.

Thank you for catching this. We have corrected this to be consistent.

*Are the proposed statistical analyses rigorous and appropriate? The use of Wilcoxon-Mann-Whitney test to compare total metastatic foci counts in the three groups is well sounded and agrees with the statistical analysis from the original paper; however, since the original paper did report raw* p*-values only, we believe it would be more appropriate to report also non-corrected* p*-values in the reviewed paper. On top of that, post-hoc methods based on the control of the FWER (such as Bonferroni) tend to be too conservative compared to methods controlling the FDR, which are the ones we believe should be used by Fiering et al. The use of Bonferroni would significantly dampen the probability to find significant differences, which we believe is somehow unfair for this Reproducibility Project.*

We have included the uncorrected tests in the analysis plan of Protocol 2 and 3. However, we do feel the use of Bonferroni to correct for multiple comparisons is appropriate. We have used an appropriately adjusted alpha error in our power calculations to account for this as these are a priori comparisons. And while FDR control provides the same degree of assurance as Bonferroni correction that there is indeed some effect, FDR is not useful to be certain that any single significant result is accurate, since some false positives are allowed, and thus is why we use a technique like Bonferroni correction, which provides a conservation control of the FWER.

On the other hand, Fiering et al. report that they will perform a meta-analysis with the results of the original paper and the new results, but there are no details about the methodology used for such a meta-analysis.

We have included in the analysis plan that we will be combining the original and replication effect sizes using a random-effects meta-analytic approach.

What can the replication team do to maximize the quality of the replication?

Please see the modifications of the protocol highlighted above. Although with 3 regions imaged per sections the authors claim to obtain a minimum power of 80%, some tumors are difficult to image (necrotic damage, autofluorescence, low fibronectin deposition or damage due to preparation or manipulation). For these reasons, we would highly recommend to stain at least two sections per tumor and to image at least five regions per section, although the more the better.

We have updated the protocol to reflect 2 sections per tumor and 5 regions per section. We have also included the exclusion criteria outlined here that might prevent a section or image from being included in the analysis.

Extracts from Reviewer #2:

*Several points not specified in* *Goetz et al. 2011* *should be clarified with the original authors, before initiating the experiments.* eLife *has verified these points with the original authors and the responses follow the questions:*

The order code of the nude mice and their age range when used for the study.

*Nude mice, official name from Harlan: Athymic Nude Mouse -Hsd: Athymic Nude-Foxn1*^*nu*^*.*

This is the order code of the nude mice in the manuscript.

And their age range when used for the study: 8-10 weeks when they arrive, about 10-12 when experiment was done.

We have updated the language to reflect the age of mice when they arrive vs. the age when the mice are injected.

*Whether DMEM with high glucose was used for* in vitro *culture of MEFs. Normal DMEM, not high glucose.*

We have updated the Materials and Reagents section to reflect this.

Original matrigel stock concentration, so the used relative dilution yields the same final concentration for implantation.

Typical protein concentrations for BD Matrigel Matrix are between 9-12 mg/ml.

This is the same Matrigel Matrix with the same concentration range listed in the Materials and Reagents section of Protocol 2. The Manufacturer is Corning, but is the same catalog and formulation as BD.

Protocol 2:

For metastasis detection, a range of exposure times from 20s to 2 min was used to detect both large and small metastases. Thus, each mouse and excised organ should be assessed to reach maximum sensitivity and count a maximum of micrometastases. To reach high sensitivity, when monitoring the lungs, liver and brain, the lower abdomen (containing tumor and luminescence in the bladder) should be shielded during longer exposure times. Also, for metastasis detection of excised organs, 2 min should be included as longest exposure time.

The maximum exposure time of 2 min is included in the procedure. The original paper only took 0.2, 1, 20, and 60 sec exposures, but we agree that increasing the potential exposure time to 2 min will enable maximum sensitivity to count micro-metastases. In correspondence with the authors the original study used only the ex vivo imaging on organs to determine the metastatic foci count. The in vivo imaging was used for assessing tumor growth and metastasis onset only.

Protocol 3:

Irrespective of the power calculation, because of expectable intra-tumor variation of stromal density and organization, several sections from the same tumor should be analyzed for fibronectin alignment and cell elongation. The analysis of only 3 fields from a single section may be flawed by accidental sample variability and insufficient representativity.

We have updated the protocol to reflect 2 sections per tumor and 5 regions per section. We have also included the exclusion criteria outlined by Reviewer #1 that might prevent a section or image from being included in the analysis.

Extracts from Reviewer #4:

For protocol 2 and 3 the exact details of what was found in the original paper are reported (generally groups means and SDs) so I easily can follow this logic of how the sample size/power calculations have been derived. I have a number of questions regarding assumptions made in these sample size calculations:

1) Often the SD used in the power calc is estimated on a group of size between 6 and 15 so these estimates will tend to be underestimates. This is not so much of a problem when the effect sizes are approximately 1 SD but it could still be taken account of. I cannot replicate the pooled SDs used in the tables – mine come out slightly larger.

We have checked our calculations, and obtained the same pooled SDs as originally reported. We are using the formula: $\sqrt{\frac{\left(\left(N_{1}-1\right)\times \left(SD_{1}^{2}\right)\right)+\left(\left(N_{2}-1\right)\times \left(SD_{2}^{2}\right)\right)}{\left(N_{1}+N_{2}-2\right)}}$

2) Sometimes the type 1 error is adjusted for multiple testing and sometimes it is not. If the same animals are studied for several outcomes then these should be treated as multiple testing problems. When several organ sites are examined this must surely be the same animals?

Thank you for catching this oversight. We performed the calculations with the alpha error of the multiple organs at 0.01 (to account for the 6 groups) instead of 0.05. As expected, the sample size we are using is not sufficient to perform the individual organ comparisons that were performed in the original paper. We have excluded these from our analysis. However, the main claim is the comparisons between the total metastatic foci count per mouse, which correctly has the alpha error adjusted.

3) It seems odd to me that the replication study wants to replicate results that were not statistically significant in the original study? In these cases they argue that they select an effect size that they have 80% power to detect.

Sometimes we are including comparisons that did not obtain a statistically significant effect in the original study to determine if we see the same non-statistically significant effect size, and because some of these effects, such as the comparison between LM-4175 cells alone and LM-4175 cells with KO MEFs, are of scientific interest to determine if they are not the same. The effect size we report is determined by the sample size, which is determined by other statistically significant effects, the alpha error, and the pre-defined power of 0.80. Thus, we are presenting what effect size could be detected, if there is one, for these comparisons.

4) There are many hypotheses being tested in this replication. Are they all equally important? Are some of them nested? If half of the tests are statistically significant will this be regarded as a validation of the original report? I am not confident that these sample sizes will have sufficient power for so many repeated tests.

We have now excluded all of the individual organ comparisons that were performed in the original paper, because our sample size is not sufficient. However, the main claim is the comparisons between the total metastatic foci count per mouse, which correctly has the alpha error adjusted. With the rest of the included tests, we have performed sample size calculations to ensure our sample size is sufficient with a power of at least 80%.

5) There is some confusion in statistical language which may be a feature of the G*power program. The sample size calculations state that t*-tests (to detect standardised effects) will be used but the analysis plan uses non-parametric tes*ts *(Kruskall Wallis and Mann Whitney, etc).*

Yes, we agree that the language can be a little confusing, however, the Kruskall Wallis and Mann Whitney are listed in the sample size calculations as the type of F, or t, non-parametric test used, similar to how Spearman’s rho is listed as a type of correlation test. G*Power lists all parametric and non-parametric tests within the same test family.

[Editors' note: further revisions were requested prior to acceptance, as described below.]

Both Abstract and Introduction should clearly state, as it was stated in the first version of this manuscript, that the 50 papers being replicated are the top 50 most impactful ones in the cancer biology field, and not just 50 random papers from this field. This critical piece of information has now been removed from both Abstract and Introduction. Also, the Abstract should state clearly that the authors are replicating not the whole paper, but only a fraction of it.

The first two sentences of the Abstract, as well as the last sentence was changed by an editorial revision on 10/23/14, with the same three sentences used to ensure consistency across the Registered Reports. This is the same language used in the published Registered Reports to date. However, if the reviewers prefer the language to include the word ‘impactful’, we defer to the *eLife* editorial board who can comment on the ability to do this for the published Registered Reports to ensure consistency.

We have also added the exemplifier ‘which are a subset of all the experiments reported in the original publication’ to the Abstract when describing the experiments outlined in the Registered Report.

Several important controls are missing: it would be important to check by WB that the pMEFs generated are actually KO for caveolin-1. Also, to make sure that WT pMEFs have increased expression of (at least) smooth muscle actin (SMA) compared to KO pMEFs, as a marker of increased activation and ECM remodeling capabilities of these pMEFs.

We agree and have updated the manuscript to include these quality controls, which were similarly reported in Goetz et al. in Figures 7CA and Supplemental Figure 2A.

If the authors wish to insist on the discrepancy between the Capozza et al. and the Goetz et al. studies, they should inject the B16F10 cells used by Capozza to clarify whether the discrepancy may be caused by a difference in cell lines.

We have edited the text in the Introduction to remove any indication of a discrepancy between the Capozza et al. and the Goetz et al. studies. We agree that the metastasis formation in this model is the primary focus of Protocol 2 and have indicated this in the Introduction as well. As an added aspect of the experimental approach, we have included the primary tumor growth data analysis, just like the original study, however the inclusion of this data is also only in respect to this exact model, not to the potential of Cav1 on primary tumor growth in other models, including the ones presented in Goetz et al. (Figures S7A-B). As such, we will restrict our analysis to the experiments being replicated and will not include discussion of experiments not being replicated in this study.

Regarding statistics, the reviewers were confused by the answer of the authors to question 3 of Reviewer #4. If the authors want to see if the effect of these distinct groupings is equivalent, it seems one should not do a superiority power calculation but rather a bioequivalence. The authors have used a 15% adjustment, which is what is done to convert numbers in a parametric calculation to a non-parametric calculation. The answer seems to imply (in protocol 2) that the authors found they needed 39 animals and adjusted this to 48). Then the authors used the sample size to see what effect size this would have 80% power to detect? However, the reviewers did not follow how the authors got to 39 animals in the first place. Could the authors clarify this point?

To clarify the earlier comments and the intent of these calculations, the detectable effect size reported for the primary tumor size analysis is not to test if the groups are equilavent, but rather to determine what effect size can be detected if there is a difference. The original study did not observe a statistically significant effect based on the sample size they used, and as the reviewer points out, this does not imply the groups are equivalent, but rather not different. The same analysis will be conducted in the replication study, but since the ability to detect a statistically significant difference is dependent on sample size we calculated the effect size that could be detected with the sample size that will be used.

To clarify the sensitivity calculation, we knew the sample size is 49 based on the total metastatic foci per mouse sample size calculation. However, since we will be performing a non-parametric test, we adjusted the sample size accordingly since we were determining the detectable effect size using a parametric calculation. We have updated this section to clarify this point. We also adjusted the numbers to reflect the detectable effect size using the average group size since 41 (or 49) are not multiples of the number of groups, which is 3. Originally we had used the next lowest sample size divisible by 3, which was 39 and 48, respectively, but since this is an estimate of the detectable effect size, the difference does not change the analysis plan. This calculation is intended to report what effect size can be detected with the sample size and test used, not the sample size needed to detect a given effect size.

*Finally, the authors should indeed report also the raw* p*-values. This is very important because corrected* p*-values (especially with Bonferroni) will always be less significant than the raw ones, so some of the significant results could be* “*lost*” *if only corrected* p*-values were reported.*

We will report both corrected and uncorrected *p*-values. We will also report the effect size and 95% confidence interval to provide another means of evaluating the data.
